# Simultaneous presentation of juvenile ossifying fibroma in the maxilla and mandible: a case report

**DOI:** 10.1016/j.ijscr.2020.05.025

**Published:** 2020-05-22

**Authors:** Vildeman Rodrigues de Almeida Júnior, Joaquim de Almeida Dultra, Paloma Souza Gonçalves Cerqueira, Tarcísio Oliveira Donato Fernandes, Flávia Caló de Aquino Xavier, Jean Nunes dos Santos, Águida Cristina Gomes Henriques

**Affiliations:** a Postgraduation Program in Dentistry and Health, Federal University of Bahia, Salvador, BA, Brazil; b Dental School, Federal University of Bahia, Salvador, BA, Brazil

**Keywords:** Ossifying fibroma, Juvenile ossifying fibroma, Fibro-osseous lesions

## Abstract

**Introduction:**

Juvenile ossifying fibroma (JOF) is a controversial and uncommon lesion that has been distinguished from the larger group of ossifying fibromas because of distinct clinical features and some morphological peculiarities. Furthermore, JOF shows an aggressive biological behavior that has led researchers to consider it a benign neoplasm, resulting in its differential diagnosis with important benign and malignant bone neoplasms.

**Presentation of case:**

This study describes a case of synchronous presentation of JOF in the mandible and maxilla of a young patient. In addition, the literature was reviewed to identify clinical-pathologic features and possible factors that could help establish the correct diagnosis. A 26-year-old male patient presented simultaneously a lesion affecting the body, angle and ramus of the left mandible and another lesion in the left maxilla. Both lesions were well delimited and radiolucent, being unilocular in the maxilla and multilocular in the mandible. The mandibular lesion was partially resected and the maxillary lesion was submitted to curettage. The diagnosis was JOF.

**Dicussion:**

A delay in seeking medical care and a late diagnosis can have serious consequences for the postoperative functional and esthetic outcome. Much care should be taken during establishment of this diagnosis since an equivocal diagnosis can have serious consequences for the patient in terms of treatment.

**Conclusion:**

After 1 year, the patient shows no signs or symptoms of recurrence of the lesions and was referred for reconstructive surgery of the mandible. An early and correct diagnosis is necessary to permit the best therapeutic management.

## Highlights

• Juvenile ossifying fibroma (JOF) has distinct clinical features and some morphological peculiarities.
• In gnathic bones, JOF occur more frequently in the maxilla.
• It is necessary to correlate clinical, radiographic and microscopic findings for the correct diagnosis of JOF.
• The aggressive biological behavior of this lesion indicates the need for an early diagnosis.

## 1 Introduction

Fibro-osseous lesions are a diverse group of pathologic conditions characterized by the replacement of normal bone tissue with fibrous tissue that contains mineralized material at different stages of mineralizaton [1]. The spectrum of these lesions includes a variety of developmental lesions, reactional processes and neoplasms such as fibrous dysplasia, cemento-osseous dysplasia (COD), ossifying fibroma, and juvenile ossifying fibroma (JOF) [2].

Juvenile ossifying fibroma is an uncommon benign fibro-osseous neoplasm that is distinguished from the larger group of ossifying fibromas based on patient age, the sites most commonly affected, and its more aggressive biological behavior [3]. According to the WHO (2017), two histopathologic variants of JOF are recognized, the trabecular (JTOF) and psammomatoid (JPOF) variant [4]. Two variants commonly affect younger patients, with a mean age of 11 years for JTOF and of 22 years for JPOF. Both variants have a male predilection. In gnathic bones, they occur more frequently in the maxilla [4], [5]. The two variants exhibit a similar growth pattern and radiographic features. Characteristics suggestive of JOF include the rapid and painless expansion of the affected bone, displacement of adjacent structures, paresthesia, asymmetry and a tendency towards recurrence, associated with a radiolucent image that can contains central radiopacities [6], [7].

Microscopically, fibro-osseous lesions are characterized by the presence of irregular bone trabeculae at different stages of mineralization amidst fibrous and cellularized connective tissue [7]. Since these lesions share many morphologic features, their differentiation is often difficult. The differential diagnosis of JOF mainly includes fibrous dysplasia, COD and conventional ossifying fibroma, as well as low-grade osteosarcoma [8]. It is therefore necessary to correlate clinical, radiographic and microscopic findings for the correct diagnosis of JOF, which will support more appropriate therapy [8].

In view of the low frequency of JOF, especially in the mandible, and the difficulties encountered during its diagnosis, this study reports an uncommon case of JOF with simultaneous presentation in the maxilla and mandible. Clinical, radiographic and microscopic features are discussed, highlighting the main criteria used for its differential diagnosis.

## 2 Case Report

**Fig. 1:**
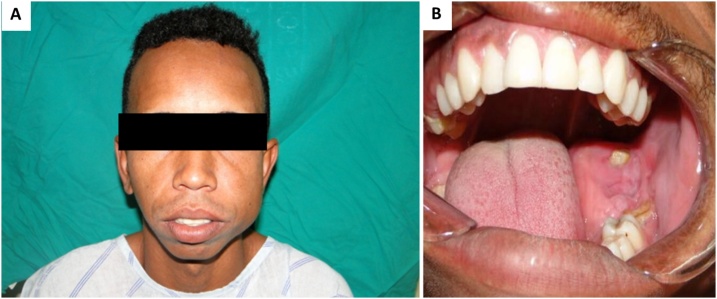
Extraoral and intraoral views. (A) Note an asymmetry in the left lower third of the face. (B) Expansion of the lingual and buccal cortical bones in the region of the left mandibular body and angle with involvement of soft tissues.

**Fig. 2:**
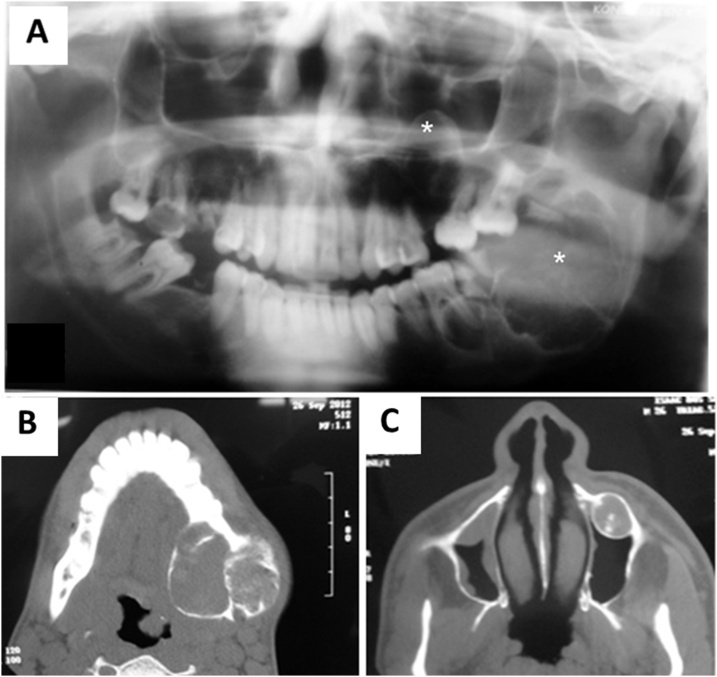
Panoramic radiograph. (A) Note two well-delimited osteolytic lesions, unilocular in the left maxilla and multilocular in the mandible (asterisk). Computed tomography. (B) Note larger diameter lesion involved the mandibular body, angle and ramus, sometimes with a characteristic of hyperdensity, which suggests areas of mineralization. (C) Note lesion in the maxilla associated with hyperdense foci and extended to the maxillary sinus.

**Fig. 3:**
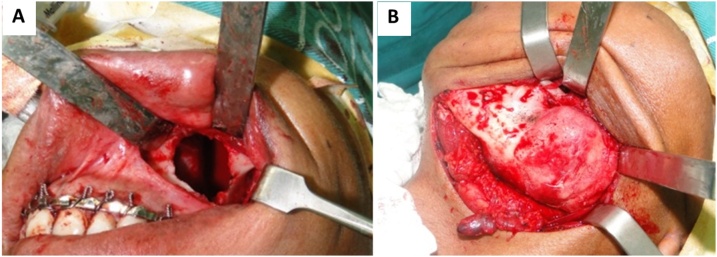
Trans-surgical approaches. (A) Curettage and peripheral osteotomy of the maxilla. (B) Resection with a safety margin in the mandibular lesion.

A 26-year-old black male patient with 2-year history of a painless swelling involving the left mandibular body. No reports of drug use, underlying diseases or harmful habits. Family history without relevant associated information. Extraoral clinical examination showed an asymmetry in the left lower third of the face (Fig. 1A). Intraoral examination identified marked expansion of the lingual and buccal cortical bones in the region of the left mandibular body and angle with involvement of soft tissues (Fig. 1B). Imaging scans revealed signs suggestive of two well-delimited and osteolytic lesions, unilocular and multilocular in the left maxilla and mandible, respectively (Fig. 2A). The lesion involved the mandibular body, angle and ramus and resulted in displacement of the affected teeth. (Fig. 2B) In the maxilla, the lesion measuring approximately 2.0 cm was associated with root resorption of the affected teeth and extended to the maxillary sinus (Fig. 2C). Pulp vitality testing of the teeth associated with the lesions was positive. The diagnostic hypotheses for the mandibular and maxillary lesions were ameloblastoma and keratocystic odontogenic tumor (KOT), respectively. An incisional biopsy was obtained from the mandibular lesion and the histopathologic diagnosis was compatible with myxoma. In view of the aggressive features of the lesions, treatment planning consisted of surgical resection with safety margins for the mandibular lesion and curettage and peripheral osteotomy for the maxillary lesion (Fig. 3A and B). Surgery performed in a specialist training program accompanied by an experienced surgeon in the field.

**Fig. 4:**
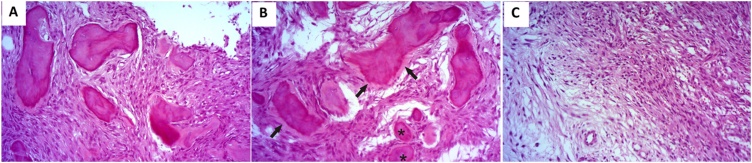
Histological sections of the mandibular JOF stained with hematoxylin-eosin. (A) Fragments exhibiting intensely cellularized fibrous connective tissue with irregular bone trabeculae at different stages of mineralization (400×, HE); (B) Spherical structures with osteoid margins (asterisk). Note trabeculae exhibiting an osteoid margin that fused with the underlying connective tissue and with brush border appearance (arrow) (400×, HE); (C) Myxoid areas intermingled with more fibrous areas in the connective tissue (400×, HE).

**Fig. 5:**
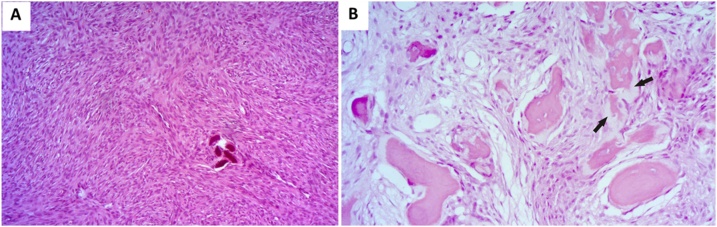
Histological sections of the maxillar JOF stained with hematoxylin-eosin. (A) Note intensely cellularized fibrous connective tissue with calcified areas (200×, HE); (B) Bone trabeculae at different stages of mineralization, exhibiting an osteoid margin (arrow) (400×, HE).

Histopathologic analysis of the two lesions revealed characteristics of JTOF. Fig. 4 shows fragments of the mandibular lesion exhibiting intensely cellularized fibrous connective tissue that contained numerous irregular bone trabeculae at different stages of mineralization (Fig. 4A) and spherical basophilic structures with osteoid margins, sometimes with a brush border appearance (Fig. 4B). Some trabeculae also had an osteoid margin that fused with the underlying connective tissue (Fig. 4B). In the connective tissue, some myxoid areas intermingled with more fibrous areas were observed (Fig. 4C). The lesion was partially lined with a fibrous capsule that separated it from normal bone tissue. The maxillary lesion exhibited characteristics similar to those described above (Fig. 5A and B). The patient is under periodic follow-up at intervals of 1 year and shows no signs or symptoms of recurrence of the lesions. The patient has now been referred for mandibular reconstruction.

## 3 Discussion

Sixteen cases of synchronous manifestation of conventional ossifying fibroma are reported in the literature [9]. On the other hand, there is only one study reporting the synchronous presentation of JOF [10], a fact highlighting the relevance of the present case.

Although JOF is a benign neoplasm, it can manifest as an aggressive lesion because of its rapid growth, expansion of cortical bones and possible displacement of adjacent structures, leading to the suspicion of a malignant neoplasm in some cases [11]. In the present case, the patient lived with the lesion for approximately 2 years before seeking medical care, which was probably due to the development of facial asymmetry.

JPOF, commonly found in the craniofacial skeleton, can affect the frontal, orbital and zygomatic bones and paranasal sinuses of young people and adults between 16 and 33 years of age. On the other hand, JTOF is a purely gnathic lesion that preferentially affects the maxilla of children aged 8 to 12 years [12], [13]. The present case was the trabecular variant of JOF and affected a 26-year-old man, an age above that commonly described in the literature. As in our case, a male predilection has been reported for both types of JOF [12], [13], [14].

The radiographic and imaging features of the present case agree with those commonly reported in the literature [2], [12], [14], [15]. According to Patigaroo [2], Urs et al. [12], Banu and Palikat [14] and Bohn et al. [15], root resorption and displacement of the affected teeth can be observed. Corroborating these findings, root resorption and tooth displacement were found in the case of the maxillary lesion, which was associated with teeth.

The clinical and imaging findings of the mandibular tumor and the rarity of JOF led to the diagnostic hypotheses of ameloblastoma, followed by KOT and myxoma. The maxillary lesion did not cause swelling or cortical bone expansion and was discovered during radiography for diagnosis of the mandibular lesion. Its radiographic appearance was suggestive of KOT. The detection of hyperdense foci in computed tomography also suggests the calcifying odontogenic cyst (COC).

The criteria available in the present case were sufficient for the diagnosis of JTOF. The lesion was characterized by intensely cellularized fibrous connective tissue associated with the presence of mineralized tissue at different stages of maturation and calcification that appeared as irregular trabeculae, some of them lined by osteoblasts [4], [7], [12].

The initial microscopic diagnosis of myxoma obtained by incisional biopsy of the mandibular lesion highlights the importance of a careful differential diagnosis. Clinical, radiographic and histopathologic findings should always be correlated since lesions with a different etiopathogenesis, behavior and prognosis can exhibit clinical and pathologic similarities. In the present case, the material collected during the incisional biopsy may have corresponded exactly to the myxoid areas found in JOF, resulting in the diagnosis consistent with myxoma.

The differential diagnosis of JOF includes fibrous dysplasia, COD, conventional ossifying fibroma, and even low-grade osteosarcoma [16], [17].

JOF have been distinguished from the large group of conventional ossifying fibroma based on the age of the patient, the site affected, and biological behavior [12]. Juvenile ossifying fibroma, either the trabecular or psammomatoid variant, affect younger patients at a mean age of approximately 11 and 22 years, respectively, while conventional ossifying fibroma is more common in the third and fourth decade of life. Both juvenile variants have a predilection for the maxilla, while the conventional type preferentially affects the mandible. Although conventional ossifying fibroma has a significant growth potential, JOF is characterized by a more aggressive biological behavior [4].

The greater cellularity and the presence of myxomatous foci and abundant osteoid material favored the diagnosis of JOF in the present study. A useful intraoperative finding that helps distinguish conventional ossifying fibroma and JOF from COD is the fact that ossifying fibromas can be separated easily from the adjacent normal bone tissue [4], as observed in the present case.

The therapeutic approach to treating JOF must consider the aggressiveness, size and potential of recurrence (30% to 58%) of this tumor. Consequently, surgical resection with safety margins seems to be the preferential treatment [8], [18]. According to El-Mofty [19], the treatment of this tumor is controversial and some authors have recommended conservative excision or curettage.

Recurrence of JOF is related to the difficulty in performing an adequate resection due to the location of the borders of the tumor and its infiltrative nature. It would therefore be more prudent to determine the therapeutic approach based on the location, extent and biological behavior of the tumor [8], [18], [19].

This study reported the synchronous presentation of a rare lesion. The aggressive biological behavior of this lesion indicates the need for an early diagnosis so that the best therapy can be instituted for the patients, preferentially conservative treatments that do not compromise function or esthetics. In the case reported a extension lesion in the mandibular body, a fact justifying the hemimandibulectomy performed. The maxillary lesion that measured approximately 5 cm and in view of the diagnostic hypothesis, a more conservative approach of surgical curettage was adopted. After 1 year, the patient shows no signs or symptoms of recurrence of the lesions and was referred for reconstructive surgery of the mandible. Despite radical treatment, the patient was well accepted with the prospect of future mandibular reconstruction.

Based on the literature review, we recognize that, despite major diagnostic difficulty, some clinical and pathologic features favor the diagnosis of JOF. Thus, the knowledge and combination of clinical, radiographic and histopathologic features of JOF are extremely important for its accurate diagnosis. The presente work has been reported in line with the SCARE 2018 criteria [20].

## Declaration of Competing Interest

We declare no conflict of interest.

## Sources of funding

This research did not receive any specific grant from funding agencies in the public, commercial or not-for-profit sectores.

## Ethical approval

Ethical approval exempted by our institution.

## Consent

Written informed conset was obtained from patient. A copy of a signed document stating this is avaliable for review by the Editor-in-Chief of this journal on request.

## Author contribution

Vildeman Almeida Júnior: performed sugery, manuscript writing, revision and submission.

Joaquim Dultra: performed sugery and manuscript writing.

Paloma Cerqueira: manuscript writing and revision.

Tarcísio Fernandes: performed sugery and manuscript writing.

Flávia Xavier: manuscript writing, revision and histopathologic analysis.

Jean dos Santos: manuscript writing, revision and histopathologic analysis.

Águida Henriques: histopathologic analysis, manuscript writing, revision and submission.

## Registration of research studies

NA.

## Guarantor

Vildeman Almeida Júnior.

Águida Henriques.

## Provenance and peer review

Not commissioned, externally peer-reviewed.
